# Global spatiotemporal distributions of lymphoma from 1990 to 2019: A Joinpoint regression analysis based on the global burden of disease study 2019, and projections until 2044

**DOI:** 10.1016/j.dialog.2024.100182

**Published:** 2024-05-21

**Authors:** Jiacheng Liu

**Affiliations:** Central South University, Changsha, Hunan China, Changsha, Hunan, China

**Keywords:** Hodgkin lymphoma, non-Hodgkin lymphoma, Cancer, Global burden

## Abstract

Lymphoma is a dissimilar collection of malignant neoplasms arising from the clonal propagation of lymphocytes. It is conventionally classified into two categories: Hodgkin lymphoma and non-Hodgkin lymphoma. The purpose of this study is to analyze the temporal patterns in the incidence of lymphoma worldwide over the past few decades and forecast the future trends from 2020 to 2044. Data on HL and NHL were obtained from the Global Burden of Disease Study 2019. In an effort to estimate the incidence rate trend, the Joinpoint regression analysis model was exploited. What's more, to project the disease burden by 2044, the Bayesian age-period-cohort analysis was employed. In 2019, higher incidence rates were observed in males and the elderly for both subtypes. Over the last three decades, a significant decline in the age-standardized incidence rate of HL was observed, while NHL has shown an increasing trend. By 2044, the age-standardized incidence rate of HL is anticipated to decrease in males and increase in females, while that of NHL is expected to rise. This study presents a new assessment of the spatiotemporal distributions of lymphoma. Significant emphasis should be placed on the effective management and long-term monitoring of patients to mitigate the potential future impact of the disease.

## 1 Introduction

Lymphoma encompasses a wide range of malignant cancers originating from lymphocytes, which includes more than 90 subtypes. Conventionally, these subtypes are divided into NHL and HL [1,2]. The clinical presentation of lymphoma is characterized by painless adenopathy, accompanied by systemic symptoms that typically arise in advanced stages [1,3,4]. Treatment approaches vary hinging on the primary subtype. HL is typically treated with radiation therapy or chemotherapy regimens like ABVD (doxorubicin, bleomycin, vinblastine, and dacarbazine) and BEACOPP (bleomycin, etoposide, doxorubicin, cyclophosphamide, vincristine, procarbazine, and prednisone) [[5], [6], [7]]. NHL is commonly treated with drugs such as CHOP (cyclophosphamide, doxorubicin, vincristine, and prednisone) with or without rituximab (R-CHOP), bendamustine, and lenalidomide [[8], [9], [10]]. Pathophysiological advances have led to a better understanding of lymphoma. However, significant healthcare resources are still required for the diagnostic evaluation, staging, and appropriate treatment. Moreover, challenges such as relapse and complications like secondary cancers continue to pose significant challenges. Therefore, long-term monitoring and treatment management are essential [11,12].

Prior epidemiological studies have extensively explored the risk factors linked to lymphoma, encompassing familial history, infections, inflammation, and other factors. Modifiable risk factors include current or previous tobacco use and obesity [13,14]. Given the advancements in people's life, it is crucial to assess the changes in lymphoma incidence. Previous inquiries into the burden of lymphoma have primarily focused on regional or national contexts or specific subtypes, providing limited insights into future disease burden [[15], [16], [17], [18], [19], [20]]. Therefore, it is essential to evaluate the disease burden of lymphoma across different subcategories.

The Global Burden of Disease (GBD) 2019 Study database was utilized to analyze the global incidence data of lymphoma from 1990 to 2019, presents a new assessment of the global spatio-temporal distributions of lymphoma and provide insights for the development of prevention strategies.

## 2 Materials and methods

### 2.1 Source of data

GBD study provides a comprehensive assessment of mortality rates, disease prevalence, and risk factors across a wide range of causes, diseases, and injuries, which encompasses 286 causes of death, 369 diseases and injuries, and 87 risk factors, spanning over 200 countries and regions [[21], [22], [23], [24]]. Data for the GBD Study was gathered from various sources such as censuses, household surveys, civil registration, vital statistics, disease registries, health service utilization, satellite imagery, disease notifications, and other resources. Previous research projects have utilized GBD datasets to validate the credibility of the data and the representativeness of the sampled populations [17,18,25]. To estimate the burden across different conditions in 204 countries and territories spanning from 1990 to 2019, researchers utilized DisMod-MR 2.1, a Bayesian meta-regression modeling tool [[26], [27], [28]]. This tool integrated all the aforementioned data sources for each disease, and a bias correction process was implemented to calculate country-specific prevalence and disease burden estimates, as documented in prior research. To account for uncertainty, uncertainty intervals (UIs) were computed by extracting the 25th and 75th percentile values from the 1000 draws of the posterior distribution [22,26]. The classification of HL and NHL was made according to International Classification of Diseases-10 (ICD-10) codes. Data from the years 1990 to 2019 were gathered from the website of GBD 2019 (http://www.healthdata.org/) [[29], [30], [31]]. Data was categorized based on sex and age groups, which encompassed the following ranges: 0–14, 15–19, 20–24, 25–29, 30–34, 35–39, 40–44, 45–49, 50–54, 55–59, 60–64, 65–69, 70–74, 75–79, 80–84, 85–89, 90–94, and 95+ years of age. The age-standardized rate for two subtypes of lymphoma is calculated on the basis of the GBD 2019 global age-standardized population [32,33].

### 2.2 Joinpoint analysis

Utilizing the temporal patterns of disease spread, Joinpoint creates segmented regression models and optimizes trend analysis for individual data points within each segment [34,35]. This approach enables a detailed exploration of unique disease variations across different time frames worldwide [[36], [37], [38]]. Additionally, the annual percentage change (APC) and the average APC (AAPC) were computed to depict the magnitude of the tendency in change [31,37].

### 2.3 Bayesian age-period-cohort projection

In order to measure temporal trend direction of lymphoma and calculate the age-standardized incidence rate of both subtypes from 2020 to 2044, a Bayesian age-period-cohort (BAPC) analysis model was conducted [25]. The BAPC model suggests that age, period, and cohort effects have comparable impacts over adjacent time intervals. Within the BAPC model, all unknown parameters are considered random, with suitable prior distributions. Bayesian inference utilizes a second-order random walk to smooth the prior effects of age, period, and cohort [[39], [40], [41]]. It has been observed to provide better coverage and accuracy compared to traditional projection models such as the Nordpred model, smooth spline model, and Poisson regression [24,38,[42], [43], [44]]. To evaluate the performance of BAPC models, the data from 1990 to 2019 were divided into two intervals (1990–2014 and 2015–2019) [45]. Subsequently, the data from 1990 to 2014 were used to predict the incidence of lymphoma from 2014 to 2019, and the observed values from 2014 to 2019 were employed to validate the accuracy of the projected figures. The error rate, calculated as (predicted values - observed values) ÷ (observed values), was used to assess the model's performance [40,45].

We operated BAPC analyses separately for males and females using the BAPC and INLA package in the R programming language. The aim of these analyses was to forecast the age-standardized incidence rates from 2020 to 2044.

## 3 Results

### 3.1 Disease Burden in 2019

**Fig. 1:**
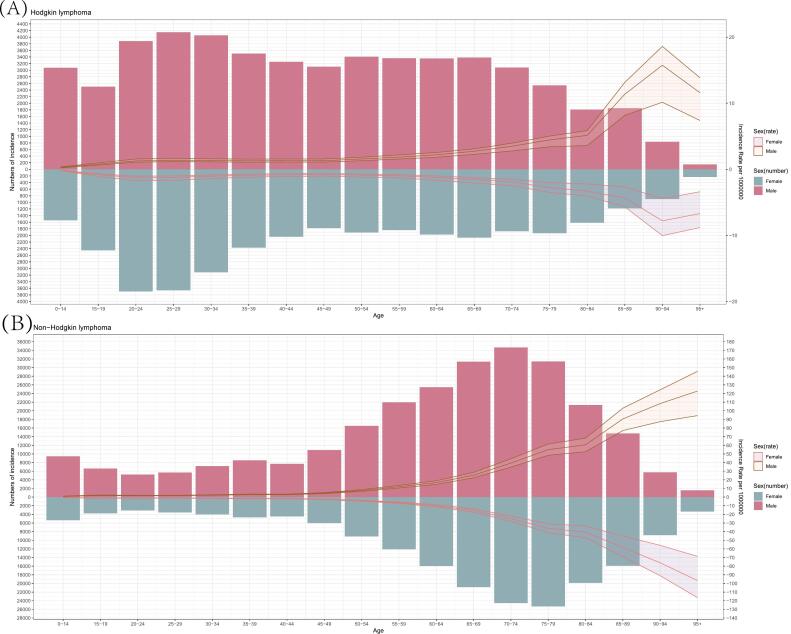
Global numbers and rates of incidence of Hodgkin lymphoma (A) and non-Hodgkin lymphoma (B) by age and sex in 2019. Shading represents the upper and lower limits of the 95% UI.

Fig. 1 showed the disease burden of lymphoma across the world in 2019. The total number of incidence cases of Hodgkin lymphoma was up to 87,508.87 with a 95% uncertainty interval (UI) of 77,941.13 to 101,425.4. This encompassed 51,302.7 cases (95% UI 43617.47 to 58,702.52) in males and 36,206.18 cases (95% UI 30171.35 to 46,093.64) in females. Furthermore, the rate was higher in men across overall age groups.

Correspondingly, in 2019, the number of incidence cases of non-Hodgkin lymphoma amounted to 457,076.5(95% UI, 416894.4 to 498,784.8), including 266,093.6 (95% UI 241381.1 to 291,086.7) in men and 190,982.9 (95% UI 169138.7 to 210,588.5) in women. Moreover, the rate remained a rising tendency with increasing age in both males and females.

### 3.2 Global Trends in lymphoma from 1990 to 2019

**Fig. 2:**
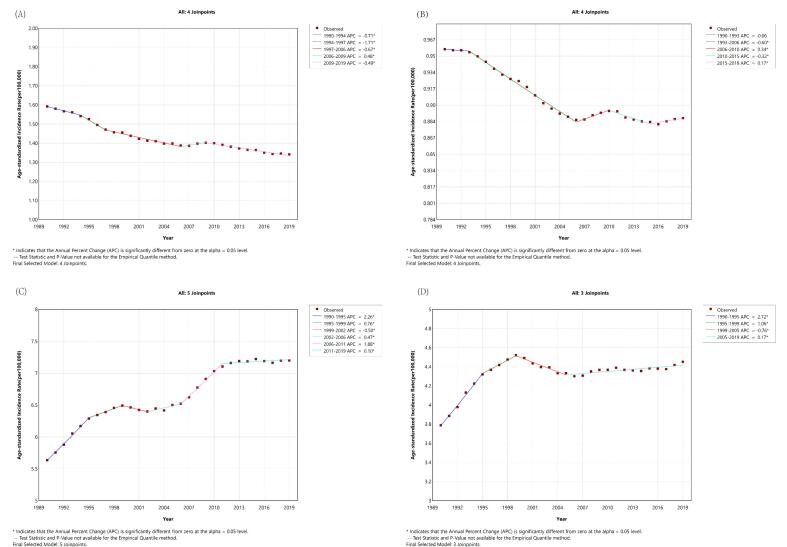
Joinpoint regression analysis of the incidence rate of lymphoma in male and female (1990–2019). (A) Hodgkin lymphoma incidence in male, (B) Hodgkin lymphoma incidence in female, (C) Non-Hodgkin lymphoma incidence in male, (D) Non-Hodgkin lymphoma incidence in female.

The curves in Fig. 2 show the outcome of Joinpoint analysis of changing trends of lymphoma. As displayed, the age-standardized incidence rate of males with HL descended from 1990 to 2019. The AAPC was −0.6034 (95% UI -0.6263 to −0.5755). The trend declined with the APC of −0.7086 (95% UI -0.8749 to −0.3690) from 1990 to 1994, −1.7114 (95% UI -1.8960 to −1.3548) from 1994 to 1997, −0.6713 (95% UI -0.7726 to −0.5651) from 1997 to 2006, and − 0.4903 (95% UI -0.5778 to −0.4312) from 2009 to 2019. Whereas there was a mild incremental increase in 2006–2009, with an APC of 0.4839 (95% UI 0.0691 to 0.6637). The incidence rate of HL in females from 1990 to 2019 was divided into five stages, with an AAPC of −0.2616 (95% UI -0.2758 to −0.2471). It decreased with an APC of −0.2471 (95% UI -0.2457 to 0.2148) in the period of 1990–1993, −0.6018 (95% UI -0.6293 to −0.5790) in 1993–2006, and − 0.3170 (95% UI -0.5146 to −0.2116) in 2010–2015. However, it increased with an APC of 0.3380 (95% UI 0.2128 to 0.4887) in 2006–2010 and 0.1693 (95% UI 0.0394 to 0.3921) in 2015–2019.

Regarding the burden of NHL, the incidence rate in men, as shown in Fig. 2C, increased from 1990 to 1995, 1995 to 1999, 2002 to 2006, 2006 to 2011, and 2011 to 2019 with an APC of 2.2621 (95% UI 2.1142 to 2.4259), 0.7559 (95% UI 0.5936 to 1.0424), 0.4674 (95% UI 0.2286 to 0.9385), 1.8801 (95% UI 1.7331 to 2.0968) and 0.1040 (95% UI 0.0003 to 0.1898), which had a descent from 1999 to 2002, with an APC of −0.5046 (95% UI -0.7018 to −0.1495). The AAPC for the overall period was 0.8550 (95% UI 0.8303 to 0.8783). Moreover, Fig. 2D shows the incidence rate of NHL in women. It increased in 1990–1995, 1995–1999, and 2005–2019 with APCs of 2.7171 (95% UI 2.4632 to 3.0140), 1.0562 (95% UI 0.6430 to 1.4262), and 0.1666 (95% UI 0.0809 to 0.2665). However, it decreased in 1999–2005 with an APC of −0.7554 (95% UI -1.2556 to −0.5448). The AAPC for this period was 0.5321 (95% UI 0.4988 to 0.5697).

### 3.3 Projections of lymphoma from 2020 to 2044

**Fig. 3:**
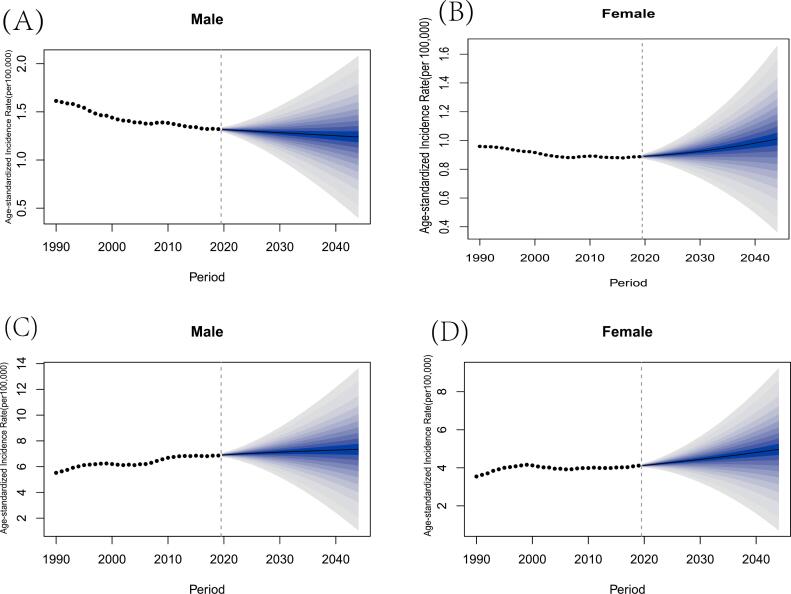
The projection of the incidence rate of lymphoma from 2020 to 2044 in male and female (A) The age-standardized incidence rate of Hodgkin lymphoma for male. (B) The age-standardized incidence rate of Hodgkin lymphoma for female. (C) The age-standardized incidence rate of non-Hodgkin lymphoma for male. (D) The age-standardized incidence rate of non-Hodgkin lymphoma for female.

Based on the data from 1990 to 2019, the BAPC model was used to project the total incident cases from 2020 to 2044. The accuracy was estimated using the data from 1990 to 2019. The outcomes of the BAPC model's projection revealed low error rates across all demographic categories (less than 5%) [45]. The projected age-standardized incidence rates of two subtypes of lymphoma worldwide from 2020 to 2044 are illustrated in Fig. 3. As shown in Fig. 3A, the age-standardized incidence rate of HL in males is estimated to steadily decrease. It is estimated to be 1.240144 per 100,000 people in 2044. What's more, the trend of the incidence rate in females, as shown in Fig. 3B, is different. It is predicted to increase to 1.010326 per 100,000 people in 2044.

When it comes to NHL, the age-standardized incidence rate shown in Fig. 3C and D is estimated to rise. The rate of males is projected to rise from 6.916939 in 2020 to 7.345737 per 100,000 persons in 2044. Similarly, the incidence rate in females is expected to increase from 4.127969 in 2020 to 4.971642 per 100,000 people in 2044.

## 4 Discussion

This study revealed the spatiotemporal distributions of lymphoma incidence cases among different age groups and genders around the world and the changing trend was also analyzed. A comprehensive evaluation of the burden of lymphoma based on more extensive and updated data is shown in this research.

The variation of the global burden of HL and NHL in 2019 was observed. The bimodal distribution of the incidence of HL was shown. The incidence rate was found to increase with advancing age for both genders. Additionally, male predominance was observed in all age groups for both subtypes. These results were generally consistent with previous studies [1,16,18,19]. However, the detailed epidemiological characteristics of lymphoma also vary among different countries [15,17]. From 1990 to 2019, the incidence rate of HL had fallen in different genders. Epstein-Barr virus (EBV) is reckoned to be etiologically associated to HL [46,47]. Though the influence of EBV has remained controversial, previous epidemiological studies have shown compelling proof of the pathogenic contribution of EBV. The temporal change in the incidence rate of HL may be associated with global development [46,[48], [49], [50]]. The age-standardized incidence rate of NHL was found to rise in both males and females. NHL includes more than 50 different subtypes, with the major part of NHL derived from B cells. The increased rate of incidence may be attributed to the advancement in early detection. Moreover, prognosis of NHL is also influenced by various factors, including histopathology, the extent of involvement, and other relevant factors [8]. The five-year survival rate for NHL is calculated to be approximately 72.0% [1,51].

The present study utilized BAPC analysis to predict the incidence rate of lymphoma from 2020 to 2044. The estimated results showed that the age-standardized incidence rate of HL of male will keep decreasing but the rate in female show an increasing trend. NHL's age-standardized incidence rate is predicted to keep rising over the next three decades. The difference in the incidence of HL the projection aligns with existing research findings. The economic status of populations has been linked to HL incidence, with women living in impoverished areas having a lower incidence of HL [20,52]. When it comes to NHL, a previous study has found that the incidence has been decreasing only in regions with high SDI over the past decades [4,20]. The global age-standardized incidence rate, as depicted in Fig. 3C and D, is estimated to increase in both genders.

Projection of the incidence rate highlights the necessity for improving global cancer prevention measures to alleviate the burden of lymphoma. Previous studies have extensively analyzed the risk factors [16,17]. These factors include aging, family history, infections, lifestyle, and immune disorders [1,13,14].

There were several limitations in this study. To begin with, it is difficult to completely avoid bias, although efforts have been made to ensure comparability, which might exert an influence on the completeness and accuracy of the data [31]. Moreover, impacts from other factors that were not included in the GBD study, such as ethnicity, cannot be completely ignored. Other social and environmental factors were also not included in the study [53,54]. Furthermore, the GBD database had shortcomings, such as issues with data quality assurance. Last, predictions of the disease burden of HL and NHL did not consider potential advancements in the disease's background, which could only reflect changes in population growth and demographic shifts [43]. Therefore, further large-scale cohort studies should be conducted to address these deficiencies and enhance the understanding of the burden of lymphoma disease.

In conclusion, this study shows a comprehensive estimate of the disease burden of lymphoma worldwide. The results show that it will be necessary to give more priority to providing better management for patients with lymphoma. The study findings might be helpful in alleviating the disease burden of lymphoma.

## 5 Conclusion

To recapitulate in short, this study presents a new assessment of the global spatio-temporal distributions of lymphoma. Males have a higher number of reported cases compared to females. The incidence rate for elderly individuals was higher in both genders. The incidence rate of Hodgkin lymphoma shows a downward trend in males and is expected to increase in females, while the rate of non-Hodgkin lymphoma demonstrates an increasing tendency. The findings indicate that health policies to alleviate the disease burden of lymphoma are still necessary.

## Ethics and consent

Ethical permission was not requested in the study in accordance with the local legislation and institutional requirements. Written informed consent from the participants' legal guardian/next of kin was not required to participate in this study in accordance with the national legislation and the institutional requirements.

## Funding statement

This research did not receive any specific grant from funding agencies in the public, commercial, or not-for-profit sectors.

## CRediT authorship contribution statement

**Jiacheng Liu:** Writing – review & editing, Writing – original draft, Visualization, Validation, Supervision, Software, Resources, Project administration, Methodology, Investigation, Funding acquisition, Formal analysis, Data curation, Conceptualization.

## Declaration of competing interest

The authors declare that they have no known competing financial interests or personal relationships that could have appeared to influence the work reported in this paper.
